# Validity, Reliability and Responsiveness of Wound‐QoL‐14 Quality of Life Questionnaire in Patients With Diabetes Related Foot Ulcers

**DOI:** 10.1111/iwj.70816

**Published:** 2026-03-08

**Authors:** L. Hitchman, F. Siracusa, R. Lathan, B. Ravindhran, J. Long, G. Smith, M. Sidapra, I. C. Chetter

**Affiliations:** ^1^ Faculty of Clinical Sciences Hull York Medical School Heslington UK; ^2^ Department of Vascular Surgery Hull University Teaching Hospitals Hull UK

**Keywords:** diabetic foot ulcers, quality of life, wound healing

## Abstract

Diabetes related foot ulcers (DFU) are associated with significant physical, psychological and social dysfunction. Measuring the impact of treatments on patients' overall well‐being is vital to ensure care is holistic. This study aimed to validate the Wound‐Qol‐14 quality of life assessment tool in people with a DFU. A single centre longitudinal prospective validation study in people with DFUs was conducted. Wound‐Qol‐14, Diabetic Foot Ulcer Scale—Short Form (DFS‐SF) and EuroQol 5 dimensions 5 levels (EQ‐5D‐5L) were completed by people with DFUs at baseline and 6 months. Wound‐Qol‐14 was repeated within 7 days of the first questionnaire. Correlation coefficients with a cut off of ≥ 0.7 were used to assess convergent validity, divergent validity and responsiveness to changes in DFU severity. Reliability was assessed using Cronbach's *α*. The study aimed to recruit 100 people. One hundred and seven people were recruited. The mean age was 62 (SD 13) years and 85 (79.4%) were male. The mean DFU duration was 30 (SD 83) days and the mean DFU area was 6.2 (SD 10.7) cm^2^. Convergent validity was demonstrated in all domains of Wound‐Qol‐14 and DFS‐SF (*r* − 0.695 to −0.799) except burden/bothered about ulcer care domains (*r* = −0.443). There was moderate correlation between Wound‐Qol‐14 domains and EQ‐5D‐5L dimensions (*r* = 0.477–0.501). Cronbach's *α* ranged from 0.683 to 0.919 for the domains of Wound‐Qol‐14. Wound‐Qol‐14 was not responsive to changes in DFU severity (*r* = −0.291; 95% CI −0.501 to −0.048) but was responsive to healing status (healed 1.14 [IQR 0.86] vs. unhealed 1.86 [IQR 1.47]; *p* = 0.017). Wound‐Qol‐14 is a valid and reliable tool to measure disease‐specific quality of life in people with DFUs. Further work is needed to refine the responsiveness. EQ‐5D‐5L should be used to measure generic quality of life in people with DFUs.

## Summary

- Wound‐Qol‐14 is valid and reliable tool to measure disease specific quality of life in people living with diabetes related foot ulcers.
- Wound‐Qol‐14 is responsive to healing status but not changes in SINBAD scores in people living with diabetes related foot ulcers.
- EuroQol 5 Dimensions 5 Levels (EQ‐5D‐5L) should be used to measure generic quality of life in people living with diabetes related foot ulcers.

## 1 Background

Diabetes mellitus is a major public health and socioeconomic concern with more than 589 million people living with the disease globally [1]. Diabetes related foot ulcers (DFU) are a common complication that occurs due to a combination of neuropathy and ischaemia [2]. An estimated 33–60 million adults living with diabetes have a DFU and the global prevalence is around 6.3%. The lifetime risk of developing a DFU is between 12% and 34% [3].

The physical, emotional and socioeconomic dysfunction caused by DFUs is significant [4, 5, 6, 7]. Patients often report that healthcare providers do not address their needs holistically, which can lead to them disengaging from services [8]. One way of assessing whether healthcare is improving outcomes that are important to patients is by using patient‐reported outcomes measures (PROMS). One patient centric outcome measure is health‐related quality of life (HRQoL) and is often assessed using self‐reporting questionnaires. These can either be generic or disease specific. One of the most frequently used generic HRQoL questionnaires is the EuroQoL 5 Dimensions 5 Levels (EQ‐5D‐5L). EQ‐5D‐5L measures five domains of health including mobility, self‐care, usual activities, pain/discomfort and anxiety/depression. Each domain is subsequently divided into five levels: no problems, slight problems, moderate problems, severe problems and extreme problems [9, 10, 11]. For people with DFUs, the disease‐specific HRQoL questionnaire is the Diabetic Foot Ulcer Scale—Short Form (DFS‐SF). It encompasses 29 questions across 6 domains: leisure, physical health, dependence/daily life, negative emotions, concern with ulcer/feet and bothered by ulcer care. DFS‐SF has demonstrated good internal consistency, reliability, construct validity and responsiveness in people with DFUs [12].

Another tool used to assess HRQoL in people with chronic wounds is Wound‐QoL‐14. Wound‐Qol‐14 has potential benefits over DFS‐SF as it is shorter to complete and therefore may result in a higher return rate and patient acceptability. The questionnaire was developed from three established disease‐specific instruments: the Freiburg Life Quality Assessment for wounds (FLQA‐w), the Cardiff Wound Impact Schedule (CWIS) and the Würzburg Wound Score (WWS) [13]. Wound‐Qol‐14 is a valid tool to measure HRQoL in people with chronic wounds and captures relevant aspects of living with a chronic wound [14, 15], but it has not been specifically validated in people with DFUs. Several studies have found Wound‐Qol‐14 can measure the interaction between wound characteristics, patient characteristics and functioning, for example, leisure [16, 17]. This supports the use of Wound‐Qol‐14 as a tool to measure HRQoL in people with DFUs in clinical and research settings if it is demonstrated to be valid. The purpose of this study was to assess the validity, reliability and responsiveness to healing of Wound‐Qol‐14 in people living with a DFU.

## 2 Methods

A longitudinal validation study of the Wound‐Qol‐14 questionnaire in patients with DFUs. The study is reported with reference to the COSMIN guidelines [18].

### 2.1 Study Design

A single centre prospective validation study of Wound‐Qol‐14 in patients with DFUs.

### 2.2 Aims

The aims of the study were to determine the validity (primary), reliability and responsiveness (secondary) of Wound‐Qol‐14 in people with DFUs.

### 2.3 Eligibility Criteria

Patients with diabetes mellitus and foot ulcer, defined as a break in the skin below the medial malleoli, were eligible to take part. Participants also had to be over the age of 18 and able to complete the questionnaires in the English language. Patients were excluded if they were not expected to live for more than 6 weeks to avoid bias from other significant health conditions.

### 2.4 Study Setting

The study was conducted in vascular and podiatry outpatient clinics at a tertiary care hospital in England.

### 2.5 Index Test

The index test was Wound‐Qol‐14. Wound‐Qol‐14 is the shortened version of Wound‐Qol‐17 which has been validated in people with lower limb wounds of various aetiologies. It comprises 14 questions and responses are rated on a 5‐point scale between ‘not at all’ to ‘very much’. It calculates disease specific quality of life in three domains: body, psych and everyday life and a global score. The lower the value the better quality of life [13, 19].

### 2.6 Reference Standard

The disease specific reference standard was DFS‐SF. The generic reference standard was EQ‐5D‐5Ls. DFS‐SF consists of 29 questions which cover six domains of health including leisure, physical health, dependence/daily life, negative emotions, concern with ulcer/feet and bothered by ulcer care. Each question is rated on a 5‐point scale between ‘none of the time’ and ‘all of the time’. A higher score equates to a better quality of life [20].

EQ‐5D‐5L measures health in five dimensions which include mobility, self‐care, usual activities, pain/discomfort and anxiety/depression. Each dimension has five levels between no problems and extreme problems. The results are mapped onto country‐specific utility values. Utility values range between 1 (*perfect health*) and 0 (*death*). The questionnaire also includes a visual analogue scale between 0 (*worse health*) and 100 (*best health*) for patients to rate how good or bad their health is on the day of completion [11].

### 2.7 Data Collection

Demographic information and clinical indicators were extracted from medical records. Demographic data included patient age, sex, body mass index, smoking status and co‐morbidities. DFU details included ulcer size, ulcer duration, SINBAD score and Wi‐Fi stage. SINBAD score, Wi‐Fi stage and DFU size were also collected 1 and 24 weeks after the first appointment.

Questionnaires were completed at the first podiatry appointment, 1 week after the first podiatry appointment and at 24 weeks. Questionnaires were self‐completed by patients in a random order. To minimise missing data, questionnaires were reviewed by a researcher to ensure the participant had selected a response to all questionnaire items.

Data were collected onto case report forms which were kept in a locked research office. The final data was transferred onto an Excel spreadsheet for cleaning before importing into SPSS for analysis.

### 2.8 Analysis

Wound‐Qol‐14 and DFS‐SF domain scores were calculated following the publishers' instructions. EQ‐5D‐5L utility values were calculated using the English value set [21, 22].

Patient demographic and DFU data were analysed using descriptive statistics. Count data is presented as counts, frequencies and percentages. Continuous data is presented as mean and standard deviation or median and interquartile range. Only completed questionnaires were analysed.

Validity is defined as the extent to which an instrument measures what it is supposed to measure [23]. This was judged by assessing construct validity, which consists of convergent and divergent validity. Convergent validity is demonstrated when two measures that are supposed to be measuring the same construct (‘like’) are highly correlated. Contrarily, divergent validity would demonstrate low correlation between different (‘non‐like’) domains. Construct validity was studied by calculating a correlation coefficient between the total and subscale scores of each questionnaire. A cut‐off of ≥ 0.7 was used to demonstrate convergent validity. Contrary to convergent validity, a distinct cut‐off for the correlation coefficient is not recommended in the literature for divergent validity; instead, questionnaires were assessed individually [24].

Responsiveness is the ability to detect a change when it has occurred in the construct the tool is measuring [25]. A criterion approach was used to assess responsiveness between Wound‐Qol‐14 and DFS‐SF and EQ‐5D‐5L. Changes in responses to like domains and global scores between Wound‐Qol‐14 and DFS‐SF and Wound‐Qol‐14 and EQ‐5D‐5L at baseline and 24 weeks were compared and a correlation coefficient was calculated. Satisfactory correlation was indicated by a correlation coefficient ≥ 0.7. A construct approach was used to explore if a change in DFU size, severity and healing status correlates with changes in QoL scores. The correlation coefficient was used to compare changes in Wound‐Qol‐14 global scores with DFU size and severity. Mann Whitney U test was used to compare Wound‐Qol‐14 global scores with healing status at 24 weeks.

Floor and ceiling effects occur when a significant proportion of participants of a questionnaire select the lowest/worst or highest/best scores, respectively. This results in the measure being unable to discriminate between these participants [26]. Common causes of this include items irrelevant to a particular condition, items which are not responsive to intervention and too broad categories [23]. Wound‐Qol‐14 responses were assessed for floor and ceiling response in the different Wound‐Qol‐14 items graphically.

Reliability is whether the test receives the same results when it is repeated on the same participants when no change in the participants' state has occurred [23]. This was assessed by examining the internal consistency and test–retest reliability. Internal consistency tests if items on a questionnaire that are measuring the same thing are highly correlated. This was calculated for Wound‐QoL‐14 domains. Cronbach's *α* was used to assess for satisfactory internal consistency, with a cut‐off of ≥ 0.7. Test–retest reliability was assessed by comparing questionnaire responses completed within 7 days of the initial questionnaire, when it was unlikely the clinical condition had changed significantly. A correlation coefficient with a cut‐off ≥ 0.7 indicated a good correlation.

All data were analysed using SPSS version 28 (Version 28.0.0; Armonk, NY, USA).

### 2.9 Sample Size

As per COSMIN guidance, the study aimed to recruit at least 100 participants [18].

### 2.10 Ethics Statement

The study was approved by the Hull University Teaching Hospitals NHS Trust audit department and the NHS Health Research Authority (HRA). It was reviewed by the Wales Research Ethics Committee (reference: 22/WA/0089; IRAS 311664). The study did not affect routine patient care. Permission to use DFS‐SF, Wound‐QoL 14 and EuroQoL‐5D‐5L was granted from the licence holders.

## 3 Results

The study recruited 107 patients between 26 May 2022 and 31 October 2023. The mean age of the cohort was 62 (SD 13) years and most of the participants were male (79.4%). The majority of DFUs were located on the forefoot and mean DFU duration was 30 (SD 83) days at the point of recruitment (Table 1). Mean Wound‐Qol‐14, DFS‐SF and EQ‐5D‐5L scores at baseline and 24 weeks are presented in Table 2.

**TABLE 1:** Patient and DFU demographics.

Demographic	*N* = 107
Person	
Age, mean (SD), years	62 (13)
Sex, *n* (%)	M 85 (79.4): F 22 (20.6)
Smoking status, *n* (%)	
None	42 (39.3)
Ex‐smoker	42 (39.3)
Current	18 (16.8)
BMI, mean (SD), kg/m^2^	31.3 (8.3)
IHD, *n* (%)	40 (37.4)
HTN, *n* (%)	67 (62.6)
COPD, *n* (%)	5 (4.7)
CKD^a^, *n* (%)	28 (26.2)
PAD^b^, *n* (%)	40 (37.3)
DFU	
Laterality, *n* (%)	Left 44 (41.1): Right 58 (54.2)
Location, *n* (%)	
Forefoot	77 (71.9)
Midfoot	14 (13.1)
Hindfoot	10 (9.3)
Duration, mean (SD), days	30 (83)
Area, mean (SD), cm^2^	6.2 (10.7)
WiFi stage, *n* (%)	
1	71 (66.4)
2	24 (22.4)
3	2 (1.9)
4	4 (3.7)
SINBAD score, *n* (%)	
0	2 (1.9)
1	13 (12.1)
2	35 (32.7)
3	33 (30.8)
4	12 (11.2)
5	3 (2.8)

Abbreviations: BMI: body mass index; CKD: chronic kidney disease; COPD: chronic obstructive pulmonary disease; DFU: diabetes‐related foot ulcer; HTN: hypertension; IHD: ischaemic heart disease; PAD: peripheral arterial disease; SD: standard deviation; SINBAD: site, ischaemia, neuropathy, bacterial infection, area, depth; Wi‐Fi: wound ischaemia foot infection.

^a^ Stages 2–5.

^b^ PAD diagnosed as ABPI < 0.8 or TBPI < 0.7.

**TABLE 2:** Wound‐Qol‐14, DFS‐SF and EQ‐5D‐5L Scores.

	Baseline, median (IQR) (*n* = 107)	24 weeks, median (IQR) (*n* = 68)
Wound‐Qol‐14		
Body	0.75 (1.25)	1 (0.50)
Psych	1.5 (2.25)	1.5 (2.25)
Everyday life	1.8 (2.40)	1.2 (1.6)
Global score	1.43 (1.72)	1.36 (1.34)
DFS‐SF		
Leisure	60 (60.00)	95 (48.75)
Physical health	65 (65.00)	92.5 (43.75)
Dependence and daily life	77.5 (50.00)	100 (37.50)
Negative emotions	66.67 (63.54)	91.67 (50.00)
Worried about foot/ulcer	56.25 (75.00)	84.375 (67.19)
Bothered about ulcer care	62.5 (50.00)	93.75 (43.75)
EQ‐5D‐5L		
Mobility	3 (2.5)	2 (2)
Self‐care	1 (1)	1 (1)
Usual activities	3 (3)	2 (2)
Pain/discomfort	2 (1.5)	2 (2)
Anxiety/depression	2 (2)	1 (2)
Utility value	0.690 (0.420)	0.807 (0.460)
Visual analogue scale score	60 (40)	71.5 (40)

### 3.1 Floor and Ceiling Effects in Wound‐Qol‐14

Questionnaires from 107 patients were analysed. Examining responses to the baseline Wound‐Qol‐14 questionnaire, most respondents selected the lower values until Item 5. This includes all the items that compose the body domain. The floor effect begins to dissipate at Item 6 and there is a wider spread in responses for the remainder of the questionnaire. A ceiling effect was not observed (Appendix A, Figures A1, A2, A3, A4, A5, A6, A7, A8, A9, A10, A11, A12, A13, A14).

### 3.2 Validity

Questionnaires from 107 patients were analysed. Convergent validity was demonstrated for most of the domains between Wound‐Qol‐14 and DFS‐SF. The Wound‐Qol‐14 domains Body, Psych, Leisure and Everyday life strongly correlated with DFS‐SF Physical Health, Negative Emotions/Worried about foot/ulcer, Leisure and Dependence and Daily Life, respectively. Wound‐Qol‐14 Item 5 (burden of DFU) was moderately correlated with DFS‐SF Bothered by DFU care domain (Table 3). Convergent validity was not demonstrated between Wound‐Qol‐14 and EQ‐5D‐5L. Domains of Wound‐Qol‐14 were moderately correlated with dimensions of EQ‐5D‐5L (Table 4).

**TABLE 3:** Convergent validity between Wound‐Qol‐14 and DFS‐SF.

Wound‐Qol‐14 domain	DFS‐SF domain	Correlation, *r* (95% CI) (*n* = 107)	*p*
Body	Physical health	−0.738 (−0.815 to 0.634)	< 0.001
Psych	Negative emotions	−0.729 (−0.809 to −0.624)	< 0.001
Psych	Worried about foot/ulcer	−0.799 (−0.860 to −0.716)	< 0.001
Everyday life	Dependence and daily life	−0.728 (−0.807 to −0.622)	< 0.001
Item 12 (leisure)	Leisure	−0.695 (−0.784 to −0.579)	< 0.001
Item 5 (burden)	Bothered about ulcer care	−0.443 (−0.587 to −0.271)	< 0.001

**TABLE 4:** Convergent validity between Wound‐Qol‐14 and EQ‐5D‐5L.

Wound‐Qol‐14 domain	EQ‐5D‐5L dimension	Correlation, *r* (95% CI) (*n* = 107)	*p*
Body	Mobility	0.477 (0.314–0.613)	< 0.001
Psych	Anxiety/depression	0.501 (0.342–0.632)	< 0.001
Everyday	Usual activities	0.489 (0.328–0.622)	< 0.001
Global score	Visual analogue scale score	−0.456 (−0.596 to −0.290)	< 0.001
Global score	Utility value	−0.488 (−0.621 to −0.329)	< 0.001

Examining divergent validity, Wound‐Qol‐14 body domain was moderately correlated with DFS‐SF negative emotions, worry about foot/ulcer, dependence and daily life, leisure and bothered about ulcer care domains. Wound‐Qol‐14 psyche domain was moderately correlated with DFS‐SF dependence and daily life domain and strongly correlated with DFS‐SF physical health, leisure and bothered by ulcer care domains. Wound‐Qol‐14 everyday life domain was moderately correlated with DFS‐SF worry about foot/ulcer domain and strongly correlated with DFS‐SF physical health, negative emotions, leisure and bothered by ulcer care domains. Wound‐Qol‐14 item 12 (leisure) was moderately correlated with DFS‐SF physical health, negative emotions, worry about foot/ulcer, dependence and daily life and bothered by ulcer care domains. Wound‐Qol‐14 item 5 (burden) was weakly correlated with DFS‐SF physical health and dependence and daily life domains and moderately correlated with DFS‐SF negative emotions, worry about foot/ulcer and leisure domains (Table 5). There was weak to moderate correlation between the domains of Wound‐Qol‐14 and dimensions of EQ‐5D‐5L (Table 6).

**TABLE 5:** Divergent validity between Wound‐Qol‐14 and DFS‐SF.

Wound‐Qol‐14 domain, *r* (95% CI, *p*)	DFS‐SF domain, r (95% CI), (*p*) (*n* = 107)					
	Physical health	Negative emotions	Worry about foot/ulcer	Dependence and daily life	Leisure	Bothered about ulcer care
Body	NA	−0.484 (−0.321 to −0.620) *p* < 0.001	−0.421 (−0.248 to −0.569) *p* < 0.001	−0.477 (−0.321 to −0.613) *p* < 0.001	−0.474 (−0.308 to −0.612) *p* < 0.001	−0.510 (−0.640 to −0.351) *p* < 0.001
Psych	−0.648 (−0.519 to −0.748) *p* < 0.001	NA	NA	−0.585 (−0.699 to −0.441) *p* < 0.001	−0.624 (−0.489 to −0.730) *p* < 0.001	−0.742 (−0.818 to −0.641) *p* < 0.001
Everyday life	−0.659 (−0.757 to −0.533) *p* < 0.001	−0.695 (−0.580 to −0.783) *p* < 0.001	−0.522 (−0.650 to −0.365) *p* < 0.001	NA	−0.767 (−0.672 to −0.836) *p* < 0.001	−0.688 (−0.570 to −0.778) *p* < 0.001
Item 12 (leisure)	−0.526 (−0.369 to −0.653) *p* < 0.001	−0.546 (−0.394 to −0.669) *p* < 0.001	−0.481 (−0.617 to −0.317) *p* < 0.001	−0.509 (−0.350 to −0.640) *p* < 0.001	NA	−0.497 (−0.335 to −0.630) *p* < 0.001
Item 5 (burden)	−0.339 (−0.220 to −0.551) *p* < 0.001	−0.431 (−0.258 to −0.577) *p* < 0.001	−0.490 (−0.327 to −0.625) *p* < 0.001	−0.335 (−0.150 to −0.497) *p* < 0.001	−0.443 (−0.271 to 0.587) *p* < 0.001	NA

**TABLE 6:** Divergent validity between Wound‐Qol 14 and EQ‐5D‐5L.

Wound‐Qol‐14 domains, *r* (95% CI), *p*	EQ‐5D‐5L dimensions, *r* (95% CI), *p* (*n* = 107)				
	Mobility	Anxiety/depression	Usual activities	Visual analogue scale	Utility value
Body	NA	0.334 (0.152 to 0.494) *p* < 0.001	0.314 (0.130 to 0.477) 0.001	−0.410 (−0.558 to −0.237) *p* < 0.001	−0.467 (−0.305 to −0.604) *p* < 0.001
Psych	0.388 (0.212– 0.540) *p* < 0.001	NA	0.299 (0.114– 0.464) *p* = 0.002	−0.371 (−0.193 to −0.525) *p* < 0.001	−0.369 (−0.193 to −0.523) *p* < 0.001
EDL	0.424 (0.253–0.569) *p* < 0.001	0.406 (0.233– 0.555) *p* < 0.001	NA	−0.405 (−0.553 to −0.231) *p* < 0.001	−0.467 (−0.603 to −0.304) *p* < 0.001
GS	0.480 (0.318–0.615) *p* < 0.001	0.485 (0.323– 0.619) *p* < 0.001	0.429 (0.258– 0.573) *p* < 0.001	NA	NA

### 3.3 Reliability

**FIGURE 1:**
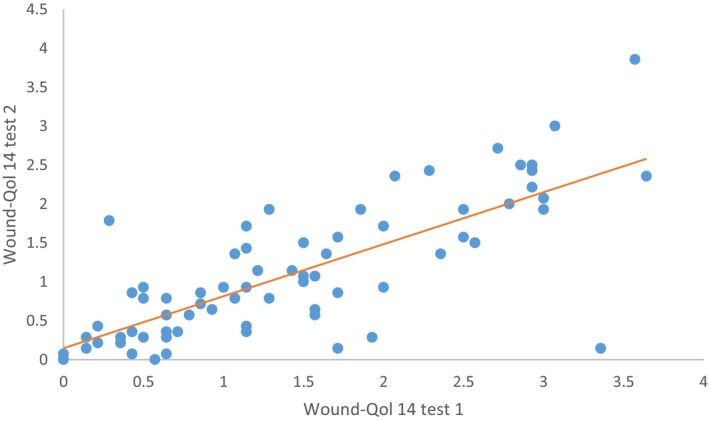
Test–retest reliability of Wound‐Qol 14. Correlation coefficient (*r*) = 0.683, *p* < 0.001.

Questionnaires from 72 patients was analysed. The test–retest reliability of Wound‐Qol 14 was 0.683 (95% CI 0.539–0.788; *p* < 0.001) (Figure 1). The internal consistency of Wound‐Qol‐14 was *α* = 0.681 for body domain, *α* = 0.868 for psych domain, *α* = 0.906 for everyday life domain and *α* = 0.919 for the global score.

### 3.4 Responsiveness

Questionnaires from 68 patients were analysed. There was a weak correlation between a change in DFU size and a change in Wound‐Qol‐14 global score at 24 weeks (*r* = 0.309; 95% CI 0.068 to 0.515; *p* = 0.013). There was a weak correlation between a change in SINBAD score and a change in Wound‐Qol‐14 global score at 24 weeks (*r* = −0.291; 95% CI −0.501 to −0.048; *p* = 0.20). There was a significant difference in Wound‐Qol‐14 global scores between people with a healed DFU and people with an unhealed DFU at 24 weeks (healed 1.14 [IQR 0.86] vs. unhealed 1.86 [IQR 1.47]; *p* = 0.017).

Comparing changes in Wound‐Qol 14 and DFS‐SF domain responses at 24 weeks, Wound‐Qol‐14 Psych domain and DFS‐SF Worried about foot/ulcer were strongly correlated (*r* = −0.700; 95% CI −0.808 to −0.545; *p* < 0.001). There was moderate correlation between changes in Wound‐Qol‐14 Psych and DFS‐SF negative emotions domains, Wound‐Qol‐14 everyday life and DFS‐SF dependence and daily life domain, Wound‐Qol‐14 item 12 (leisure) and DFS‐SF leisure domain and Wound‐Qol‐14 item 5 (burden) and DFS‐SF bothered by ulcer care domain. There was a weak correlation between changes in Wound‐Qol‐14 body domain and DFS‐SF physical health domain (Table 7).

**TABLE 7:** Changes in Wound‐Qol 14 responses compared to changes in DFS‐SF responses at 24 weeks.

Wound‐Qol‐14	DFS‐SF	*r* (95% CI) (*n* = 68)	*p*
Body	Physical health	−0.355 (−0.557 to −0.113)	0.005
Psych	Negative emotions	−0.579 (−0.724 to −0.384)	< 0.001
Psych	Worried about foot/ulcer	−0.700 (−0.808 to −0.545)	< 0.001
EL	Dependence and daily life	−0.541 (−0.697 to −0.337)	< 0.001
Item 12 (leisure)	Leisure	−0.563 (−0.715 to −0.361)	< 0.001
Item 5 (burden)	Bothered about ulcer care	−0.441 (−0.625 to −0.211)	< 0.001

## 4 Discussion

This study found Wound‐Qol‐14 to be a valid tool to measure disease specific quality of life in people living with DFUs, when compared to DFS‐SF. Wound‐Qol‐14 was not found to be a valid tool to measure generic quality of life in people with DFUs when compared to EQ‐5D‐5L. This may be because the questionnaires were measuring disease impact on quality of life differently, quality of life was assessed over different periods of time and the unmeasured impact of other comorbidities.

People living with DFUs were less affected by items that made up the body domain of Wound‐Qol‐14. This includes problems with wound pain, wound smell, discharge from the wound and effect of the wound on sleep. DFUs are most commonly neuropathic and, unless infection is present, are unlikely to have significant exudate [3]. This likely explains the floor effect observed. Wound‐QoL‐14 domains of leisure and ulcer care did not correlate with DFS‐SF like domains. This could be because single items are not sensitive enough compared to multiple items to assess these domains. However, the Wound‐Qol‐14 domain of Psych did strongly correlate with DFS‐SF Negative Emotions and Worried about foot/ulcer care domains, suggesting it was able to measure both these domains with fewer questions. Wound‐Qol‐14 showed good internal consistency for all except the body domain, suggesting it is a reliable tool in this population.

There were strong correlations between non‐like domains of Wound‐Qol‐14 and DFS‐SF, suggesting poor discriminant validity. Wound‐Qol‐14 Psych domain and Everyday life domains strongly correlate with other non‐like domains of DFS‐SF. While some of the correlations are expected, such as the correlation between Wound‐Qol‐14 everyday life domain and DFS‐SF leisure domain, the other correlations are not. This could be because Wound‐Qol‐14 has fewer items and domains than DFS‐SF; therefore, it is capturing wider constructs of health and is less discriminant than DFS‐SF.

The responsiveness of Wound‐Qol‐14 in people living with DFUs was variable. The tool was not responsive to changes in severity of DFU but was responsive to healing status. Other generic and disease specific quality of life tools have demonstrated that severity of DFU does impact quality of life, not just healing status [27, 28, 29]. This suggests that Wound‐Qol‐14 may lack responsiveness to changing disease state in people with DFUs.

Although Wound‐QoL‐14 has not been specifically validated in patients with DFUs, patients with DFUs were included in the validation studies and a large cross‐sectional study reported that wound aetiology did not significantly impact physical and psychological subscales of Wound‐QoL [30]. However, this study has found that people with DFU do respond differently to physical subscales. One of the drawbacks with Wound‐QoL‐14, which is likely to be relevant for other disease‐specific quality of life questionnaires, is that it does not mitigate the impact of other co‐morbidities. This was explored in the US Wound Registry where the median quality of life score of people with DFUs, measured using Wound‐QoL, was 0.65. This decreased to 0.49 if they had 2 or more co‐morbidities; the mean number of comorbidities per person was 8 [31]. The type of comorbidity also impacted the score and over 10 items in Wound‐QoL‐14 are impacted by other comorbidities [31]. This suggests that Wound‐QoL‐14 is limited in its ability to measure wound‐related health status independent of general health status. There is conflicting evidence on the impact of patient characteristics on HRQoL measured using Wound‐Qol‐14 [16, 32]. This emphasis the importance of validating Wound‐QoL‐14 in a population with exclusively DFUs to be able to interpret HRQoL measured using this tool.

This study has limitations. The study was conducted in a single centre in England. This could mean that results are not generalisable to other settings; however, the local population was representative of those with DFUs [33, 34]. 64% of patients completed Wound‐Qol‐14 at 24 weeks, reducing the available data to assess responsiveness. In addition, the evidence underpinning the validation of DFS‐SF has been judged to be low quality and further work is needed to determine responsiveness [35]. However, DFS‐SF remains the most developed disease specific quality of life tool for this population and therefore the most appropriate questionnaire against which to test Wound‐Qol‐14.

This study found that Wound‐Qol‐14 is a valid and reliable tool to measure disease specific quality of life in people with DFUs, responsive to healing but not changes in DFU severity. Further work to develop the responsiveness of Wound‐Qol‐14 is required before it can be considered a suitable alternative to DFS‐SF. EQ‐5D‐5L should remain the tool of choice to measure generic quality of life in this patient cohort.

## Funding

L.H. is funded by the National Institute of Health and Social Care Doctoral Research Fellowship NIHR301807. The views expressed are those of the authors and not necessarily those of the NIHR or the Department of Health and Social Care.

## Ethics Statement

The study was approved by the Hull University Teaching Hospitals NHS Trust audit department and the NHS Health Research Authority (HRA) (IRAS: 311664). It was reviewed by the Wales Research Ethics Committee (reference: 22/WA/0089). The study was sponsored by the Hull University Teaching Hospitals NHS Trust. Permission to use DFS‐SF, Wound‐QoL 14 and EuroQoL‐5D‐5L was granted from the licence holders.

## Conflicts of Interest

The authors declare no conflicts of interest.

## Figures and Tables

**FIGURE A1:**
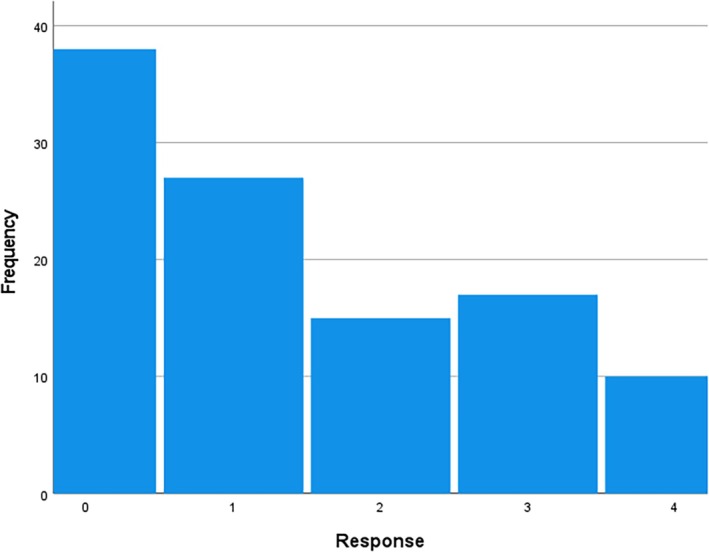
Responses to Wound‐Qol‐14 Item 1: ‘In the last 7 days my ulcer hurt’. Response: 0 = *not at all*, 1 = *a little*, 2 = *moderately*, 3 = *quite a lot*, 4 = *very much*.

**FIGURE A2:**
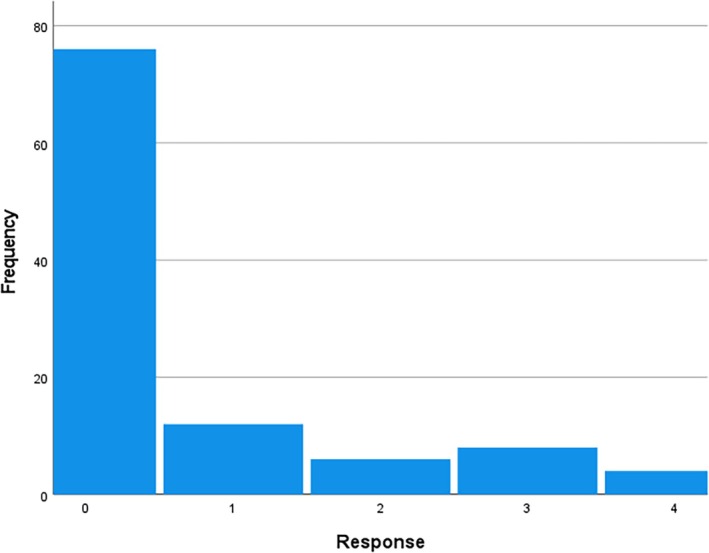
Responses to Wound‐Qol‐14 Item 2: ‘In the last seven days my wound had a bad smell’. Response: 0 = *not at all*, 1 = *a little*, 2 = *moderately*, 3 = *quite a lot*, 4 = *very much*.

**FIGURE A3:**
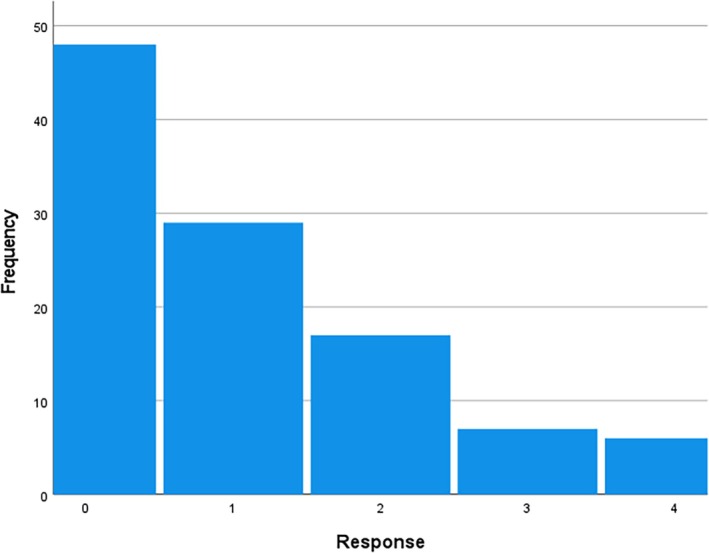
Responses to Wound‐Qol‐14 Item 3: ‘In the last seven days there was a disturbing discharge from the wound’. Response: 0 = *not at all*, 1 = *a little*, 2 = *moderately*, 3 = *quite a lot*, 4 = *very much*.

**FIGURE A4:**
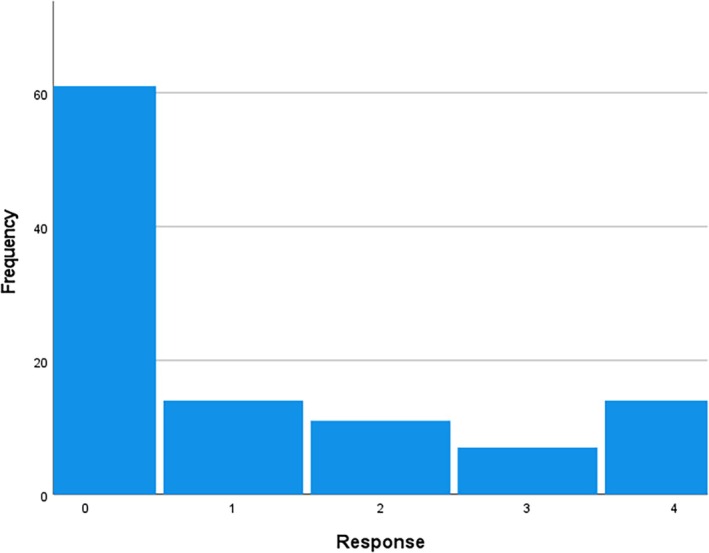
Responses to Wound‐Qol‐14 Item 4: ‘In the last seven days the wound has affected my sleep’. Response: 0 = *not at all*, 1 = *a little*, 2 = *moderately*, 3 = *quite a lot*, 4 = *very much*.

**FIGURE A5:**
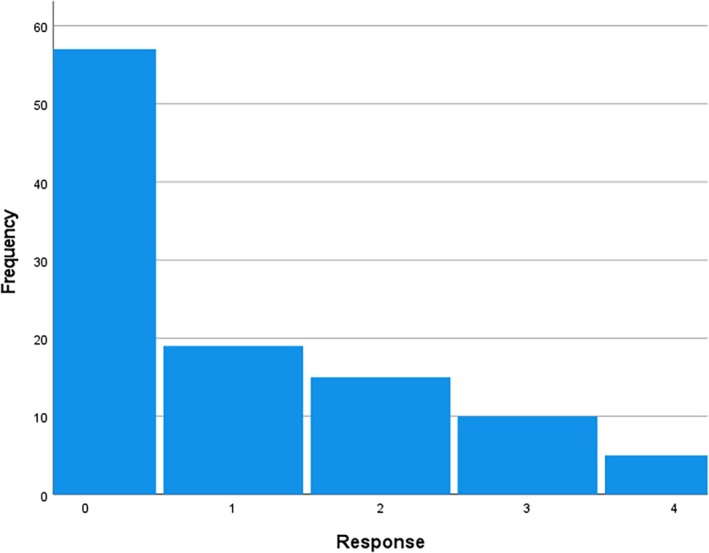
Response to Item 5: ‘In the last seven days the treatment of the wound has been a burden to me’. Response: 0 = *not at all*, 1 = *a little*, 2 = *moderately*, 3 = *quite a lot*, 4 = *very much*.

**FIGURE A6:**
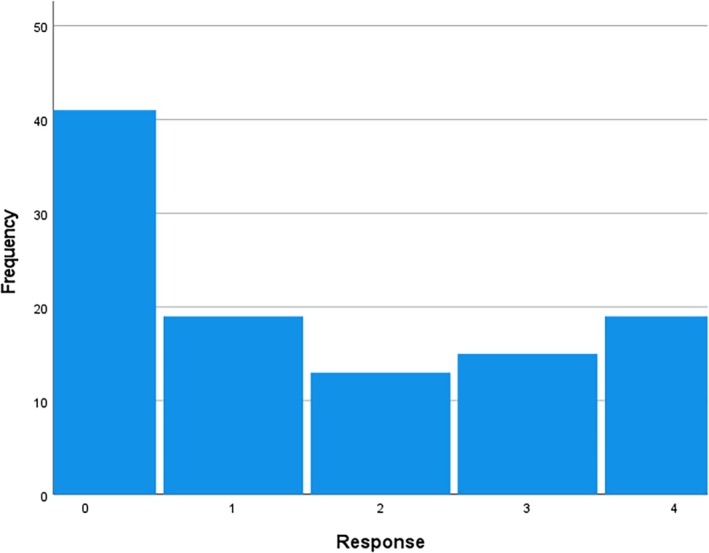
Response to Item 6: ‘In the last seven days the wound has made me unhappy’. Response: 0 = *not at all*, 1 = *a little*, 2 = *moderately*, 3 = *quite a lot*, 4 = *very much*.

**FIGURE A7:**
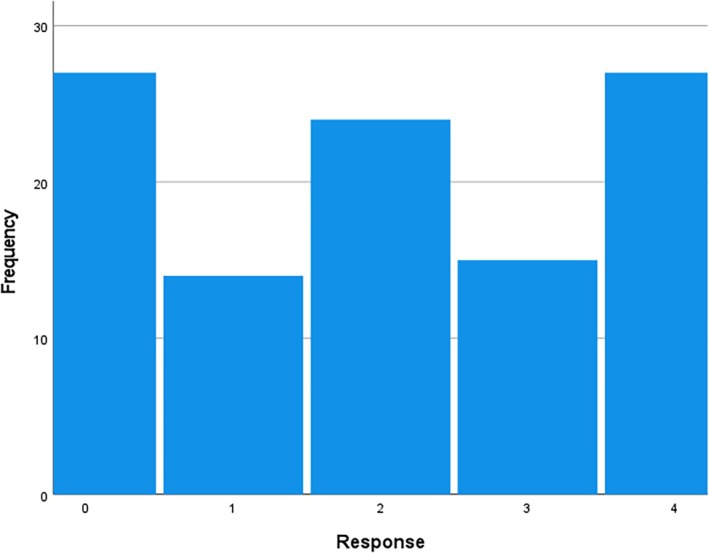
Response to Wound‐Qol‐14 Item 7: ‘In the last seven days I have felt frustrated because the wound is taking so long to heal’. Response: 0 = *not at all*, 1 = *a little*, 2 = *moderately*, 3 = *quite a lot*, 4 = *very much*.

**FIGURE A8:**
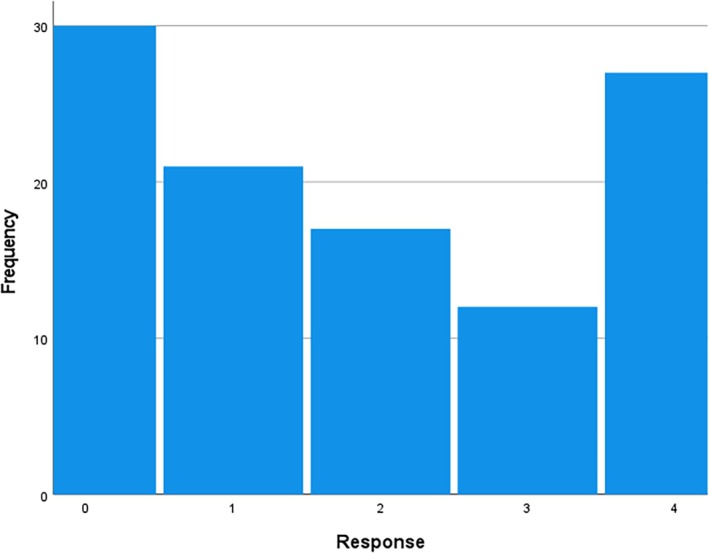
Response to Wound‐Qol‐14 Item 8: ‘In the last seven days I have worried about my wound’. Response: 0 = *not at all*, 1 = *a little*, 2 = *moderately*, 3 = *quite a lot*, 4 = *very much*.

**FIGURE A9:**
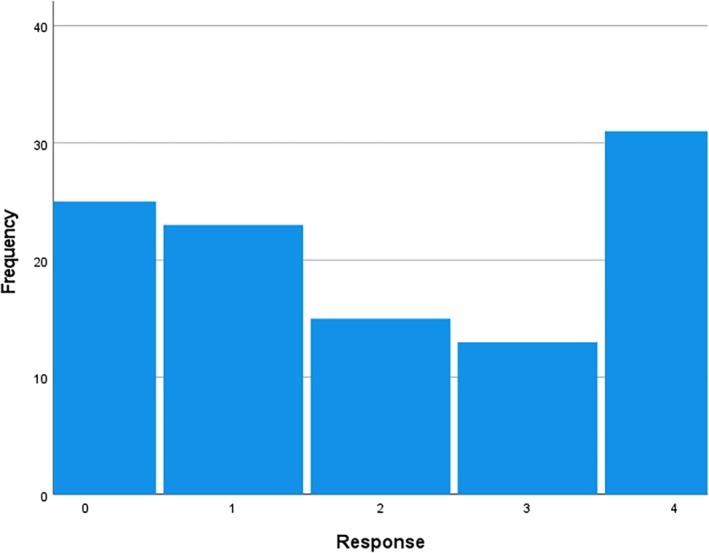
Response to Wound‐Qol‐14 Item 9: ‘In the last seven days I have been afraid of the wound getting worse or of new wounds appearing’. Response: 0 = *not at all*, 1 = *a little*, 2 = *moderately*, 3 = *quite a lot*, 4 = *very much*.

**FIGURE A10:**
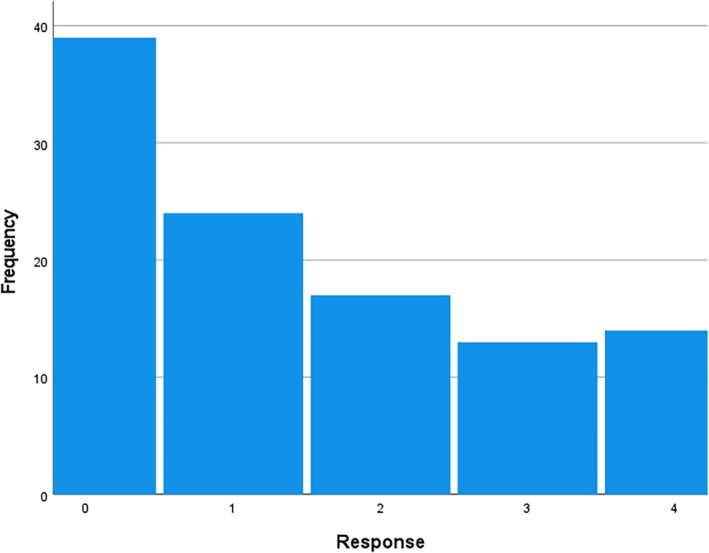
Response to Wound‐Qol‐14 Item 10: ‘In the last seven days I have had trouble moving about because of the wound’. Response: 0 = *not at all*, 1 = *a little*, 2 = *moderately*, 3 = *quite a lot*, 4 = *very much*.

**FIGURE A11:**
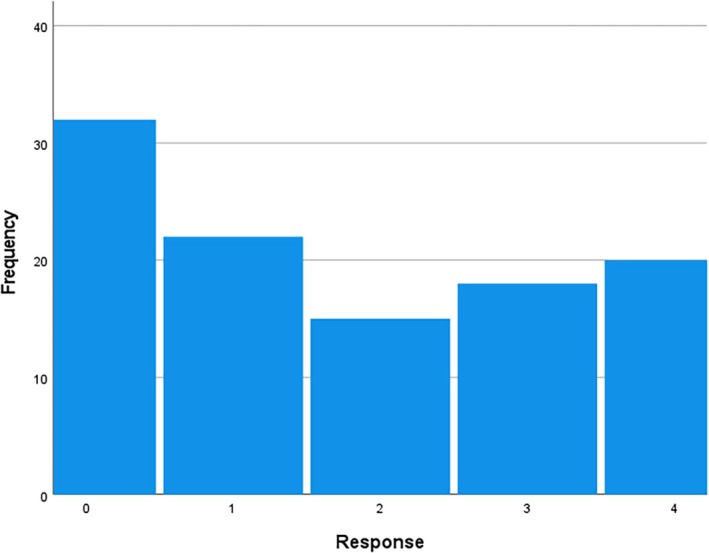
Response to Wound‐Qol‐14 Item 11: ‘In the last seven days I have had trouble with day‐to‐day activities because of the wound’. Response: 0 = *not at all*, 1 = *a little*, 2 = *moderately*, 3 = *quite a lot*, 4 = *very much*.

**FIGURE A12:**
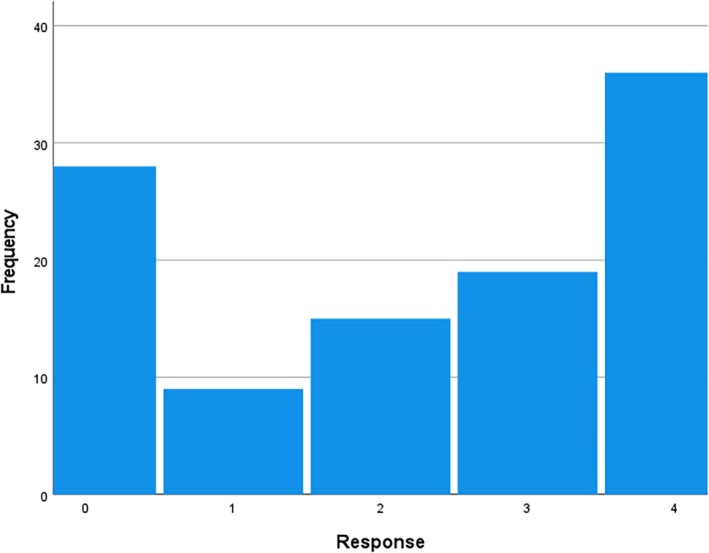
Responses to Wound‐Qol‐14 Item 12: ‘In the last seven days the wound has limited my leisure activities’. Response: 0 = *not at all*, 1 = *a little*, 2 = *moderately*, 3 = *quite a lot*, 4 = *very much*.

**FIGURE A13:**
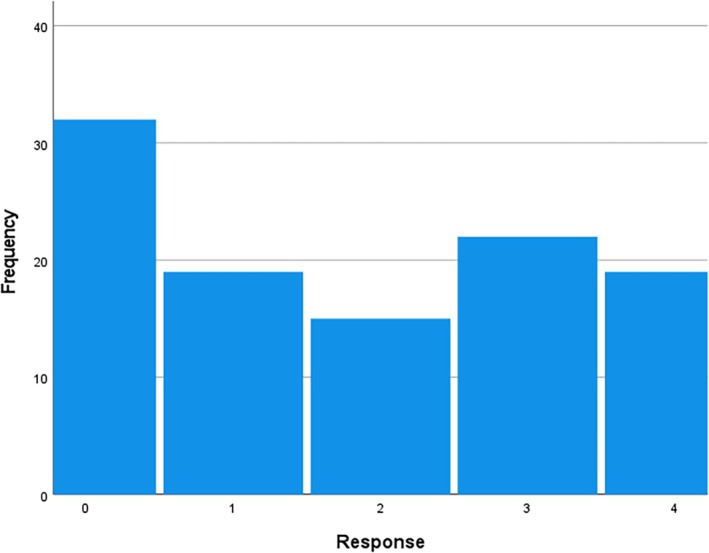
Responses to Wound‐Qol‐14 Item 13: ‘In the last seven days the wound has forced me to limit my activities with others’. Response: 0 = *not at all*, 1 = *a little*, 2 = *moderately*, 3 = *quite a lot*, 4 = *very much*.

**FIGURE A14:**
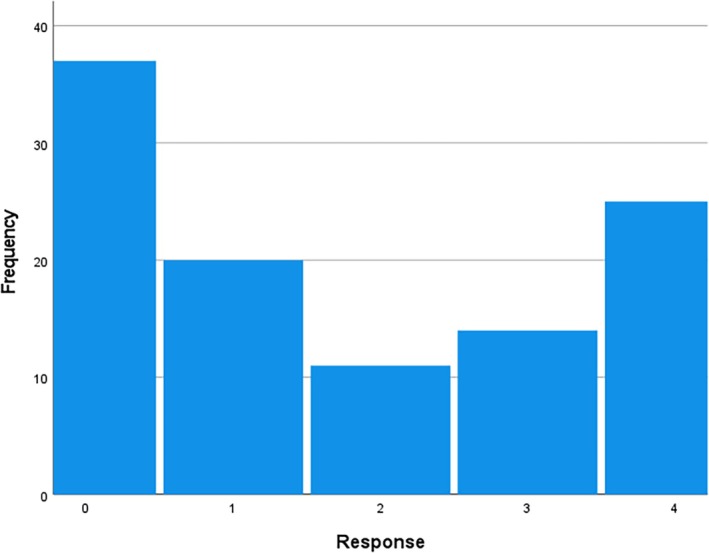
Responses to Wound‐Qol‐14 Item 14: ‘In the last seven days I have felt dependent on help from others because of the wound’. Response: 0 = *not at all*, 1 = *a little*, 2 = *moderately*, 3 = *quite a lot*, 4 = *very much*.

## Data Availability

The data that support the findings of this study are available from the corresponding author upon reasonable request.
